# SAHRANG: Subarachnoid Hemorrhage Recovery and Galantamine - A pilot multicenter randomized placebo-controlled trial

**DOI:** 10.21203/rs.3.rs-6198782/v1

**Published:** 2025-04-01

**Authors:** Bosco Seong Kyu Yang, Jude Savarraj, Elena Moreno, Kevin Immanuel, Georgene Hergenroeder, Glenda Torres, Jung Hwan Kim, Sophie Samuel, Claudia Pedroza, James Grotta, Andrew Barreto, Huimahn Alex Choi

## Abstract

**Background** Subarachnoid hemorrhage (SAH) causes life-long neurologic dysfunctions. Peripheral inflammatory processes as a reaction to brain injury has been shown to worsen outcomes after SAH. Galantamine has been shown to reduce proinflammatory microglial activities and improve synaptic connections. We hypothesize that galantamine treatment after SAH mitigates inflammation-mediated neuronal injury and improve outcomes. We conducted a pilot clinical trial to examine the tolerability and safety of galantamine in SAH patients. **Methods** This prospective, multicenter, double-blind, randomized, placebo-controlled study contiguously screened and enrolled adult patients presenting with aneurysmal subarachnoid hemorrhage of the Fisher grade 3 within 72 hours of symptom onset. A total of 60 patients were enrolled with a 1:1 ratio to two treatment arms. The first 30 patients were randomized to galantamine 8mg every 12 hours or a placebo, and the other 30 patients to either galantamine 12mg every 12 hours or a placebo. All medications were started within 36 hours after securing the aneurysm and continued for 90 days. Primary outcomes—tolerability as assessed by the number of patients who stop study medication due to adverse events associated with the study drug and mortality due to the study drug—were assessed at 90 days. **Results** There were no differences in tolerability and safety between the two groups. Bradycardia was the most common adverse event (37%), followed by clinical seizure (3%) and skin rash (3%). One subject in the galantamine group discontinued medication due to a skin rash, and another subject from the placebo group discontinued due to nausea (p=0.92). Mortality did not differ between the two groups. At 90 days, one subject from the galantamine group and four subjects from the placebo group died (p=0.34). **Conclusions** Galantamine and placebo did not differ in their side effects and safety profiles when administered to SAH patients during the early and subacute stages of the disease.

## Introduction

Subarachnoid hemorrhage (SAH) is caused by sudden rupture of a cerebral aneurysm. Although SAH represents only 10% of strokes, mortality is high (up to 35%), and morbidity is significant as patients are often young and are left with permanent cognitive and functional deficits [1]. The exact mechanism linking the initial insult to eventual comorbidity remains elusive, focusing scientific attention on molecular and cellular pathophysiology [2].

Uncontrolled inflammation is an essential component of this mechanism, supported by both clinical and animal studies [5,6]. Recent preclinical studies have suggested the importance of nicotinic α7 receptors in regulating inflammation by demonstrating that stimulation of the receptors reduces the levels of inflammatory cytokines [7]. Galantamine is an acetylcholinesterase inhibitor (AChEi) with the ability to modulate α7 nicotinic receptors allosterically and was approved in 2001 by the FDA for the treatment of mild to moderate Alzheimer's disease [8]. Galantamine has been found to be effective in improving cognitive outcomes in vascular dementia and psychiatric and motor functions in Parkinson's disease[9,10]. Among available AChEis, Galantamine has shown the strongest long-term treatment effect on cognitive functions [11]. Recent studies suggest the anti-inflammatory property of galantamine has beneficial pleiotropic effects [12]. Preclinical studies show that galantamine decreases pro-inflammatory cytokines, such as IL-1b, IL-6, and TNF-a, resulting in better hippocampal neuronal survival and improved functional outcome in pre-clinical studies [13].

In addition to these conventional roles of galantamine in neurodegenerative diseases, more recent studies have suggested that galantamine can ameliorate neurological symptoms from acute brain injury. Galantamine has been shown to improve episodic memory, alleviate depressive symptoms in patients with mild traumatic brain injury, and improve chronic aphasia due to remote ischemic stroke [14,15]. The effect of galantamine in SAH patients has not been studied. We conducted a pilot clinical trial of galantamine in SAH patients. We examined the tolerability and safety of galantamine given within 36 hours of SAH onset to up to 90 days post-hospitalization, its preliminary efficacy on functional and cognitive outcomes, and its influence on pro-inflammatory cytokines. This study has been registered at ClinicalTrials.gov under registration no. NCT02872857.

## Methods

*Study design*

This was a prospective, multicenter, double-blind, randomized, placebo-controlled study in adult patients with acute aneurysmal SAH. The aim was to investigate safety, tolerability, and preliminary efficacy of galantamine in improving long-term outcomes of acute SAH patients. The study spanned over 36 months from 2016 September to 2019 July. Patients were followed for 90 days post-enrollment. As primary safety outcomes, adverse events and mortality from study products were monitored. An interim analysis was conducted after 30 subjects were enrolled to confirm tolerability and safety. This study's protocol, informed consent documents, and any subsequent modifications were reviewed and approved by the University of Texas Health Science Center at Houston institutional review board and ethics committee. The study met all requirements, including protection of human subjects and a data safety and monitoring board (DSMB), and followed CONSORT guidelines for randomized pilot trials [16].

*Subjects*

Consecutive subjects admitted and hospitalized at the Memorial Herman Hospital System and the Methodist Hospital System in Houston, Texas, USA, were screened for inclusion if they met the following criteria: spontaneous SAH, presentation to hospital within 72 hours of symptoms, age 18-75, Fisher grade 3 hemorrhage (thick subarachnoid clot) on initial CT scan, Hunt and Hess grade 1-5 at time of randomization, presence of a cerebral aneurysm on CTA or Angiogram for which clipping or coiling is possible, and having the ability to receive medication within 36 hours of securing an aneurysm. Subjects were excluded if they had SAH due to other causes (including trauma, arteriovenous malformation, mycotic aneurysms, Moyamoya), renal disease (defined by a creatinine clearance of less than 9mL/min), history of severe hepatic impairment (Child-Pugh score of 10-15), history of COPD or asthma, co-morbid conditions likely to complicate therapy (including clinically significant arrhythmia, AIDS, autoimmune disease and malignancy), expected mortality within 72 hours, inability to complete long-term follow-up, severe functional disabilities before hospital admission for SAH [defined by pre-admission modified Rankin Score (mRS) >1], pre-existing systemic diseases which would impact long term outcomes (including stroke, myocardial infarction, pulmonary disease requiring home oxygen, chronic renal failure necessitating hemodialysis and malignancy), history of dementia, documented neurologic and psychiatric disorders, or were prisoners or pregnant women. Upon enrollment, demographic and clinical information were collected. This study was designed as a pilot study for planning of larger future studies, so it aimed to enroll a sample size deemed feasible during the study period, which was 60 subjects.

*Randomization*

To balance the severity of SAH between the control and treatment groups, we used a 1:1 ratio permuted block randomization. The Hunt and Hess Score (HH) is a score based on neurologic examination that represents the severity of SAH, and is a strong predictor of mortality after SAH [17]. Patients who present with HH 4 or 5 are at a much higher risk of dying compared to patients who present with HH1, 2, or 3. To account for this, we permuted blocks of two to four, stratifying based on admission HH (HH2 and 3 versus HH4) to ensure that groups are balanced with respect to clinical severity. Randomization was performed by research pharmacy.

*Intervention*

All patients were cared for in the Neuroscience intensive care unit by experienced neurosurgeons and neurointensivists and received standard of care per American Heart Association guidelines for treating SAH patients.

In the first phase of the trial, patients were randomized to galantamine 8mg every 12 hours or placebo and were started within 36 hours after securing the aneurysm. At the end of the first phase, an interim analysis for tolerability and safety was performed. After the tolerability and safety were confirmed, patients were randomized into either galantamine 12mg every 12 hours or placebo. For dosage adjustments, if more than nine antiemetic agents were given within 48 hours or a serious adverse event occurred, the principal investigator (PI), the treating physician, and the treating clinical pharmacist convened and determined whether the dosage should be decreased. The dosage adjustment criteria are based on our retrospective examination of 141 patients admitted with SAH. We found that 80% of the patients received anti-emetic medications with a median dose requirement of 4 doses (IQR 2-9). Thus, the 75% quartile of 9 doses was used as a screening tool to detect possible intolerability of galantamine. If it was decided that the dose should be decreased, the dose was decreased by 4 mg from 8mg to 4 mg or from 12mg to 8 mg. This occurred while all the associated personnel were blinded to treatment allocation. The PI asked the research pharmacy to decrease the dose, and the dose was decreased, regardless of whether the treatment allocation was galantamine or placebo. Once a tolerable dose was found, the subject was maintained on the dose for a total of 6 doses, and an attempt to increase the dose by 4 mg per dose was made until we reached the target dose of 8 mg twice a day in the first phase or 12mg twice a day in the second phase. If two attempts were made to reach the target dose but tolerability issues persisted, the subject remained on the tolerable dose until the end of the 90-day period.

*Safety outcomes*

Throughout the study, patients were closely monitored for potential side effects of the medication. The monitored adverse effects included arrhythmias, gastric ulcers, urinary retentions, seizures, and nausea and vomiting. Adverse outcomes were examined with the Common Terminology Criteria for Adverse Events (CTCAE) Version 4.0. When an adverse event occurred, causality was examined. All serious adverse events meeting Grade 4 criteria were communicated to the DSMB. All serious adverse events (SAE) that were unexpected and related to the intervention were reported to the DSMB within 7 calendar days of the determination by telephone, email, or fax. All other SAEs were collected and submitted with the report to the DSMB. Meetings with DSMB were held before trial initiation, and subsequent meetings were held after the first 30 subjects completed the 90-day mRS.

*Efficacy outcomes*

The primary efficacy outcome was mRS at 90 days post ictus. mRS was measured at the time of discharge, 30, 60, and 90 days post ictus. The Montreal Cognitive Assessment (MoCA) score and the EuroQOL 5-level EQ 5D (EQ5D5L) self-rated health status at 90 days were secondary outcomes for cognitive functions and quality of life (QOL), respectively. Cognitive outcomes were measured at 30, 60, and 90 days post-ictus, and a baseline cognitive status was measured with MoCA, either at discharge or 14 days post-admission. MoCA scores of 26-30 show normal cognitive function, whereas scores 0-17 indicate severe cognitive impairment. The EQ5D5L participant responses rate 5 dimensions by 5 levels of severity with higher levels representing more severe problems.

*Ancillary outcomes*

We assessed the levels of pro-inflammatory markers (central and systemic inflammation) across both arms as ancillary outcomes. Serum and cerebrospinal fluid (CSF) samples were collected at eight specified intervals depending on the type of samples: within 24 hours of admission, 24–48 hours, days

3–5, days 6–8, over 8 days but prior to discharge, and at 30, 60, and 90 days post-ictus. In total, 456 blood samples were obtained from 60 subjects, and 188 CSF samples were obtained from 55 subjects.

For plasma samples, blood was withdrawn from existing lines or by venipuncture and collected into two pink top K2 EDTA 6ml Vacutainers per time point (36 ml). The samples were placed on ice and were centrifuged at 1,459 X g for 10min at 4°C. The supernatant solution was removed and centrifuged at 1,459 X g for 10min at 4°C to generate platelet-poor plasma. Plasma was divided into aliquots and frozen at -80°C until needed. For CSF samples, sample collection required patients to maintain an admitted state, so they were collected at only five time points before their discharge. A fresh 1-5 ml sample of CSF was obtained from the ventriculostomy burette-type drain receptacle at each of five time points. The receptacle was emptied into the collection bag to start with an empty receptacle, and several drops of CSF were allowed to drip into the chamber. The drain was re-clamped; the chamber port was cleaned with chlorhexidine as per hospital specifications, and the CSF sample was drawn from the port with a sterile syringe. CSF was centrifuged (4°C, 1,459 X g for 10 minutes), portioned into aliquots, and frozen at -80°C; any residual sample was frozen at -80°C for future study. The CSF pellet was covered in a 10% DMSO solution (e.g., 100µl CSF plus 11µl DSMO) and frozen at -80°C and transferred to -145°C after 24hrs when possible.

We measured the levels of inflammatory cytokines using a 17-plex premixed immunological multiplex assay (HCYTMAG-60K-PX38, MilliporeSigma, Billerica, MA). Analyzed cytokines included Eotaxin/CCL11, PDGFABBB, MCP1, GCSF, sCD40L, MIP1a, GMCSF, IL1RA, MIP1b, IFNg, IL6, RANTES, IL10, IL8, TNFa, PDGFAA and IP10. All experiments were performed according to the manufacturer’s protocol. The levels of all markers are reported as pg/ml.

*Statistical analysis*

A prespecified safety interim analysis was performed after 30 patients were enrolled and before proceeding to the second phase of the study. Safety outcomes were dose tolerability defined as either the patient dropping out of the study because of side effects, the physician discontinuing the medication because of side effects, or occurrence of serious adverse effects (SAE) possibly related to the medication. Both outcomes were analyzed with Bayesian logistic models including treatment group and HH grade (stratifying variable) as covariates. A neutral prior center at an odds ratio (OR) of 1.0 with a 95% CI of 0.33-3.0 (Normal distribution with a mean of 0 and SD of 0.57 in the log OR scale) was used for the treatment effect. All other priors were neutral and weakly informative. The decision rule for decreasing the starting dose would be met if either (1) the galantamine group had more than a 30% rate of intolerability; or (2) there was a > 90% posterior probability of increased rate of patients with at least one in SAE in the galantamine arm compared to the placebo arm. As for efficacy outcomes, since this study was a pilot and feasibility trial, no sample size calculation or power analysis was conducted.

Continuous variables are reported as mean ± standard error (SE). Dichotomized and categorical variables are reported as proportions, while Fisher’s exact test and Wilcoxon rank-sum tests were used accordingly to test statistical significance. Incidence of primary outcomes, tolerability and mortality, was compared between the two treatment groups with Fisher's exact test. For efficacy outcomes at each time point, distributions of mRS were compared with the Chi-squared test with continuity correction, and MoCA scores, EQ5D5L VAS, and EQ5D5L indices were compared using the Wilcoxon rank-sum test. Changes in functional, cognitive, and QOL outcomes over time between assessments were calculated as a difference in outcome measures between contiguous time points. Two post hoc analyses were conducted to analyze the effects of galantamine on EQ5D5L VAS. First, the relationship between treatment group assignment and changes in individual components of EQ5D5L were analyzed, adjusting for age, sex, ethnicity, the HH grade, and disease stages. Second, the interaction between treatment groups and disease stages was analyzed by adding an interaction term. Both models used a fixed effect linear regression model to account for between-subject variations. Ancillary outcomes, including incidence of delayed complications and plasma levels of inflammatory cytokines at different time points were also compared using Fisher's exact test and Wilcoxon rank-sum test, accordingly. A conventional threshold of 0.05 was used for *p*-value to define statistical significance. All statistical analyses were performed using open-source software packages in R (v4.3.3).

## Results

From October 2016 to April 2019, we consecutively screened 377 SAH patients for eligibility (Figure 1). 315 patients were excluded, including 19% who did not have an aneurysm, 16% who were enrolled in other trials, 13% who were older than the inclusion criterion, and 12% who were expected to die within 72 hours. 72 subjects were approached for consent, and 60 subjects were randomized to either the galantamine or the placebo group (Figure 1). Two subjects from the galantamine group and another two subjects from the placebo group were lost to follow-up, however, all four subjects were confirmed to have returned to neurological baseline and were back at work by 2 months. The last-observation-carried-forward-method was used to impute the 90-day outcomes for subjects who missed the in-between follow-ups.

The average age of the study participants was 51, and 70% were female. 80% of the participants had a history of hypertension (Table 1). There were no differences in age, sex, ethnicity, and race across the two groups (Table 2). Since the subjects were randomized to the group based on clinical severity at admission, there were no differences in the HH or the World Federation of Neurosurgical Societies Scale (WFNS) score. There were no differences in the location of the aneurysms or modes of treatment to secure aneurysms across the two groups (Table 2).

*Primary Tolerability and Safety outcomes*

An interim analysis revealed no statistical difference in primary safety outcomes between the two treatment arms. In both groups, at least one subject with an SAE was observed, and the overall rates of intolerability were < 30% in both groups. The posterior probability of an increased rate of SAE in the group with a higher observed incidence rate of SAEs compared to the other group was 72%, which was lower than the preset stopping rule.

The final analysis showed no significant differences in the incidence of any adverse events across the two groups. 135 and 145 adverse events were observed in 29 and 30 patients in the galantamine and the placebo group, respectively, with the relative risk of 1.05 (95%CI: 0.83 – 1.33) comparing the galantamine group to the placebo group (Table 3). The risk of any SAE, with the relative risk of 1.33 (95% CI: 0.8 – 2.22) was not different between the groups (Table 3). We found no differences in the incidence of adverse events, including death, clinical seizures, bradycardia, tachycardia, pneumonia, hyponatremia, and anemia. The galantamine group had fewer incidences of pulmonary edema and pulmonary embolism, but the difference did not reach significance ($p > 0.05$) (Table 3). The two groups also were not different in the risk of any adverse events or any SAE directly attributable to the investigational product (Table 4).

The two treatment arms were not different in terms of mortality. At the time of discharge, one patient from the placebo arm has died. At 90 days post ictus, one patient in the galantamine and four patients in the placebo had died. The differences in mortality at both time points did not reach statistical significance ($p > 0.05$) (Table 3).

*Secondary efficacy outcomes*

At 90 days post ictus, 17 (57%) in the galantamine and 18 (60%) patients in the placebo arms achieved mRS < 3 with the median mRS of 2 for both groups. The difference did not reach statistical significance ($p > 0.05$) (Figure 2, Table 5). A higher proportion of patients achieved mRS of 0 or 1 in the galantamine group (40%) in comparison to the placebo group (20%), but the difference lacked statistical significance. Distributions of mRS were not different between the two intervention arms at other time points—at discharge, 30 days, and 60 days post ictus (Figure 2). Examining differences in changes over time, we found that although the galantamine group consistently showed more improvement in a higher proportion of patients achieving improvement in their functional status during an early phase, we were underpowered to achieve statistical significance. Between hospital discharge and 30 days post ictus, 43.3% and 33.3% of the patients showed improvement of mRS in the galantamine and the placebo groups, respectively (Figure 3). Similarly, between 30 days and 60 days post ictus, functional improvement was observed in 46.7% and 30% of the patients in the two intervention arms (Figure 3). Despite the difference in the proportion of patients who achieved functional improvements, the magnitude of improvement did not differ between the two arms. For the first, second, and third 30-day intervals, the galantamine group's mRS improved by 0.37, 0.53, and 0.27 on average, while the placebo group's mRS improved by 0.27, 0.4, and 0.3 (Supplementary Figure 1). Yet, the differences in the proportion and magnitude of functional improvements did not reach statistical significance ($p > 0.05$) (Figure 3, Supplementary Figure 1).

The two treatment groups did not differ in cognitive outcomes assessed with MoCA scores. Average MoCA scores measured at 30, 60, and 90 days post ictus were 11, 12.7, and 14 for the galantamine group and 13.2, 12.7, and 15.8 for the placebo group (Table 5, Supplementary Figure 2). For both groups, the majority of cognitive improvements occurred in the earlier phase—between discharge and 60 days post ictus—and the magnitude of cognitive improvement was also higher during the earlier stage. MoCA score improved by 3.9 and 4.42 points between discharge and 30 days post ictus for the galantamine and placebo groups, respectively, while the magnitude of improvement was 0.8 and 0.7 at the later stage (Supplementary Figure 3, 4). No statistical differences were found in all the analyzed aspects of cognitive outcome.

Patients in the galantamine group expressed significantly greater improvement in their quality of life measured with the EQ5D5L VAS scores during the early phase (between Day 30 and Day 60) in comparison to the placebo group. On average, the EQ5D5L VAS scores improved by 10.7 points in the galantamine group, while the average score decreased by 1.04 points in the placebo group ($p$-value $< 0.05$) (Supplementary Figure 5, 6, 7). Improvement in QOL at the later stages of the disease was higher in the galantamine arm but did not reach significance. Each arm showed 6.1 and 5.8 points increase in QOL. In the galantamine group, consistently higher proportion of patients achieved improvements in QOL, with 57%, between 30 and 60 days post ictus, and 53%, between 60 and 90 days post ictus, expressing improved QOL, while 36% and 52% expressed improved QOL in the placebo group. However, the difference did not reach statistical significance ($p > 0.05$) (Supplementary Figure 6, 7). Despite these differences in the temporal changes in QOL between the two intervention arms, the absolute EQ5D5L VAS values and EQ5DL indices did not differ at any time point (Table 5, Supplementary Figure 5).

Post hoc analysis showed that the galantamine group was associated with statistically significant improvement in EQ5D5L VAS scores even after adjusting for each subject's age, sex, ethnicity, and the HH grade (Supplementary table 1). The galantamine group was associated with 10.48-point ($p < 0.05$; 95% CI: 3.07 – 17.90) higher interval improvement in EQ5D5L VAS scores for a given 30-day period. Interaction analysis did not reveal any interactions between the treatment arms and disease stages (Supplementary table 1).

*Ancillary outcomes*

Two treatment arms did not show any difference in the levels of analyzed cytokines in both serum and CSF compartments (Supplementary figure 8).

## Discussion

This is a pilot clinical trial to assess the safety, tolerability and preliminary efficacy of Galantamine for the treatment of SAH in the acute and chronic stages. Galantamine was found to be safe and well tolerated. The overall rate of intolerability was similar in both arms and did not show differences in the incidence of adverse events and mortality. The galantamine and placebo group did not show differences in mRS and MoCA scores measured at all disease stages—30, 60, and 90 days post ictus. Although the absolute EQ5D5L VAS scores were not different between groups, subjects in the Galantamine group showed greater improvement in EQ5D5L VAS scores over time. Two groups did not differ in the plasma and CSF levels of inflammatory cytokines at any time points.

Galantamine is a widely used, competitive and reversible AChEi that can modulate post-synaptic nicotinic receptors. Several studies have reported the incidence of adverse events, represented by gastrointestinal symptoms, to be higher in galantamine than other AChEi like donepezil [18]. The incidence of observed adverse events is dose-dependent, especially high incidence during the initiation phase, and decreases as the treatment period extends [19]. For nausea and vomiting—the most commonly reported adverse events—the incidence rates are up to 23% within 6 months of starting the treatment and decrease to 6% after 24 months [19]. Galantamine is generally considered a well-tolerated agent, with higher than 85% of tolerability[20]. A relatively high incidence of bradycardia observed in our study should be noted, contrasting the current literature reporting its incidence to be lower than 3% [19]. As bradycardia was seen in similar proportion in the placebo arm, it is likely a complication from SAH and not from treatment with galantamine [21].

This is the first study to examine the impact of galantamine on mRS and MoCA in SAH patients. SAH survivors are subject to 2.6 times higher risk of dementia, and more than half of the survivors fail to return to their premorbid social roles [22]. Several studies report that AChEi improves memory functions in traumatic brain injury patients, but their actual effects have not been rigorously examined [23,24]. In SAH, only one study examined the efficacy of AChEi targeting cognitive using rivastigmine, but the study was performed in the chronic setting, was underpowered and lacked randomization [25]. The present study has extensively examined galantamine's side effect profiles and impact on functional and cognitive outcomes in SAH. In particular, this study has established the between-group differences in various functional outcome measures, including mRS, MoCA, and EQ5D5L VAS and index values, at multiple time points, which could serve as a prior in designing future studies. For planning of future studies if mRS at 90 days is to be used as the efficacy outcome measure, we estimate enrolling 255 patients in each treatment arm to achieve 80% statistical power at alpha of 0.05 to examine the superiority of galantamine over placebo.

Despite a limited statistical power due to its nature as a pilot study, this study suggests that galantamine could offer more improvement in EQ5D5L VAS scores over time compared to placebo. Lack of efficacy in terms of other outcome measures should be taken into account while considering the inherent limitations of the measures. Minimal detectable changes in MoCA have been reported to be as large as 5 points [26]. Although galantamine has been shown to improve cognitive deficits, the absolute magnitude of the treatment effect of galantamine has never been substantial, with the representative clinical trial showing 2.7-point changes in the ADAS-cog scale, out of 70 total points, at 6 months post-treatment [27]. As this was a pilot study, we were underpowered to detect these differences. Extended clinical trials have reported the cognitive benefit of galantamine to last as long as two years after the initiation, with a peak cognitive function observed between three to six months after the treatment [20,28]. Yet, SAH and neurodegenerative diseases show substantially different clinical courses, alluding to different drug-disease relationships. The observed clinical benefit of galantamine in terms of quality of life might be explained by the fact that the clinical benefits of galantamine have not been confined to cognitive functions—studies report galantamine has resulted in an overall improvement in the activities of daily living, behavioral disturbances, and caregiver stress [29,30]. Potentiation of multiple neurotransmitters through allosteric modulation of nicotinic receptors and enhanced thalamofrontal projection resulting in improved frontal lobe functions have been hypothesized to underlie these benefits [30]. This global improvement in neurologic functions might have served as the basis of differential improvement in EQ5D5L VAS scores.

In contrary to our expected findings, pro-inflammatory cytokines did not show differential expression between the treatment arms. Our pre-clinical study showed galantamine can activate downstream JAK2/STAT3 and IRAK-M pathway, reduce systemic inflammation, and improve behavioral outcomes in mice [7]. Systemic anti-inflammation through stimulating α7 nAChR receptors has also been shown to improve outcomes in preclinical models of other systemic inflammatory diseases like sepsis [31]. These benefits are hypothesized to result from galantamine's ability to enhance cholinergic stimulation through the vagus nerve [32]. However, studies often report depressed sympathetic tone in SAH patients [33]. It is considered our body's effort to tame the autonomic storm and catecholamine toxicity resulting from extravasation of hemolytic products and disruption of the blood-brain barrier [34]. Evidence of enhanced vagal tone or depressed sympathetic tone in SAH is also evident in the present study with unusually high rate of bradycardia. The absence of difference in cytokines might have resulted from the lack of additional benefit from further depressing sympathetic tone when the system is already in a depressed state.

This study has important limitations. First, as the study was originally designed as a pilot study, this study is not powered to draw any conclusions about the clinical efficacy of galantamine. Findings with respect to functional, cognitive, and quality of life outcomes can serve as a prior for future studies, but neither superiority nor non-inferiority can be concluded. Second, this study focused only on patients with relatively severe SAH with the Fisher grade 3. This restrictive inclusion criteria limits the generalizability of our findings. Exclusion of patients with mild SAH might have resulted in underestimating the incidence rate of complications or efficacy of the treatment. Also, the relationship between galantamine and tolerability, safety, and outcomes from patients with most severe SAH remains unknown. Third, follow-up periods were relatively short. Studies report the duration of post-SAH functional and cognitive complications to be longer than our current understanding [35]. Cognitive impairment due to SAH is observed to last as long as 2.75 years—limited by the length of observation—and the actual impact might be much longer-lasting [36]. Considering the potential benefits of long-term galantamine in other diseases, longer treatment might have resulted in a different finding.

## Conclusion

SAH causes life-long morbidity, including functional and cognitive impairment leading to poor quality of life. Galantamine holds potential to ameliorate the damage. This multi-center, double-blind, randomized, placebo-controlled pilot clinical trial investigated tolerability and safety of galantamine in SAH.

Galantamine was well-tolerable and safe to administer in SAH patients during early and late stages of the disease. Further studies are necessary to validate the tolerability and safety of galantamine and investigate its efficacy in SAH.

## Declarations

**Declarations and Statements**

The authors confirm that the manuscript complies with all instructions to the authors.

All authorship requirements have been met, and the final manuscript was approved by all authors.

This article has not been submitted or published elsewhere and is not under consideration by another journal.

The CONSORT checklist was used to ensure that the current study meets reporting standards (Schulz, Kenneth F et al. "CONSORT 2010 Statement: Updated guidelines for reporting parallel group randomised trials." Journal of clinical epidemiology vol. 63,8 (2010): 834-40. doi:10.1016/j.jclinepi.2010.02.005)

**Ethics approval and consent to participate**: This study adhered to the ethical guidelines and was approved by the Committee for the Protection of Human Subjects at The University of Texas Health Science Center at Houston, Houston, Texas (IRB No: HSC –MH-17-0452).

**Disclosure / conflict of interest:** HAC has received consultant fees from Grace Pharmaceuticals. Otherauthors have no conflict of interest to report.

**Consent for publication**: The authors provided consent for publication.

**Availability of data and material**: The data that support the findings of this study are available from the corresponding author upon reasonable request.

**Acknowledgements**: None

**Funding**: Huimahn Alex Choi received support from the National Institute of Neurological Disorders and Stroke under award (1R61NS119640-01A1 and R01NS131469) and the CCTS program at UT Health Houston.

**Author contributions:**

Bosco Seong Kyu Yang : conceptualization, methodology, formal analysis, investigation, writing – original draft, writing – review & editing, visualization

Huimahn Alex Choi: conceptualization, writing – review & editing, visualization, funding source, administration, supervision

Jude PJ Savarraj, Elena Moreno, Glenda Torres, Claudia Pedroza: methodology, validation, formal analysis, data curation, visualization

Kevin E Immanuel, Georgene Hergenroeder, Jung Hwan Kim, Sophie Samuel, James C Grotta, Andrew Barreto: validation, writing – review & editing, supervision

## Tables

Table 1
Baseline characteristics of overall population

| | **Total (n = 60)** |
|---|---|
| Age (mean, sd) | 51 (12) |
| Female (n, %) | 42 (70%) |
| Hispanic (n, %) | 33(55) |
| BMI (mean kg/m2(sd)) | 28(6) |
| OSH transfer (n, %) | 46(77) |
| Hypertension | 48(80) |
| Hyperlipidemia (n, %) | 14(23) |
| Tobacco, (n, %) | 30(50) |
| Cannabis, (n, %) | 6(10) |

BMI - Body Mass Index
OSH - Outside Hospital Transfer

Table 2
Baseline characteristics stratified by treatment arms

| | | Galantamine | Placebo |
|---|---|---|---|
| Age | | 48.3 (12.6) | 54.2 (10.4) |
| Sex (n, %) | Female | 19 (63.3) | 23 (76.7) |
| Ethnicity | Hispanics | 17 (56.7) | 16 (53.3) |
| Race (original) | Black | 8 (26.7) | 4 (13.3) |
| | White | 20 (66.7) | 25 (83.3) |
| | Other | 2 (6.6.3) | 1 (3.3) |
| HH at randomization | 2 | 7 (23.3) | 3 (10.0) |
| | 3 | 9 (30.0) | 12 (40.0) |
| | 4 | 10 (33.3) | 9 (30.0) |
| | 5 | 4 (13.3) | 6 (20.0) |
| WFNS grade on arrival | 1 | 9 (30.0) | 3 (10.0) |
| | 2 | 6 (20.0) | 10 (33.3) |
| | 4 | 4 (13.3) | 7 (23.3) |
| | 5 | 11 (36.7) | 10 (33.3) |
| Worst WFNS grade in 24 hours | 1 | 4 (13.3) | 1 (3.3) |
| | 2 | 5 (16.7) | 6 (20.0) |
| | 4 | 4 (13.3) | 8 (26.7) |
| | 5 | 17 (56.7) | 15 (50.0) |
| GCS | | 8.0 (5.0–13.8) | 8.5 (5.2–13.0) |
| Aneurysm Location | LICA | 2 (6.7) | 2 (6.7) |
| | BA | 0 (0.0) | 1 (3.3) |
| | RVA | 2 (6.7) | 0 (0.0) |
| | LPICA | 0 (0.0) | 2 (6.7) |
| | RPICA | 2 (6.7) | 0 (0.0) |
| | Pericallosal | 1 (3.3) | 0 (0.0) |
| | RSCA | 1 (3.3) | 0 (0.0) |

| | | Galantamine | Placebo |
|---|---|---|---|
| | RICA | 1 (3.3) | 2 (6.7) |
| | L.ant.choroidal | 0 (0.0) | 1 (3.3) |
| | LACA | 0 (0.0) | 1 (3.3) |
| | LMCA | 3 (10.0) | 3 (10.0) |
| | RMCA | 1 (3.3) | 3 (10.0) |
| | ACom | 8 (26.7) | 7 (23.3) |
| | Lpcom | 4 (13.3) | 3 (10.0) |
| | Rpcom | 5 (16.7) | 5 (16.7) |
| Clipping | No | 21 (70.0) | 18 (60.0) |
| | Yes | 9 (30.0) | 12 (40.0) |
| Coiling | No | 9 (30.0) | 11 (36.7) |
| | Yes | 21 (70.0) | 19 (63.3) |
| Parenchymal hematoma | No | 11 (68.8) | 12 (92.3) |
| | Yes | 5 (31.2) | 1 (7.7) |

Table 3
Incidence of adverse events stratified by treatment arms

| All Adverse Events | Galantamine (n = 30) | | | Placebo (n = 30) | | | | |
|---|---|---|---|---|---|---|---|---|
| | Total Events | Patients (n) | Mean (SD) | Total Events | Patients (n) | Mean (SD) | RR (95% CI) | p-val |
| Any Adverse Event | 135 | 29 | 4.5 (2.5) | 145 | 30 | 4.8 (2.7) | 1.05 (0.83–1.33) | 0.7 |
| Any Serious Adverse Event | 26 | 17 | 0.87 (0.97) | 36 | 17 | 1.2 (1.5) | 1.33 (0.80–2.22) | 0.27 |
| Adverse event resulting in discontinuation of IP (investigational product) | 1 | 1 | 0.03 (0.18) | 1 | 1 | 0.03 (0.18) | 0.86 (0.05–14.06) | 0.92 |
| Death during hospital stay | 0 | 0 | - | 1 | 1 | 0.03 (0.18) | - | - |
| Death at 90 days | 1 | 1 | 0.03 (0.18) | 4 | 4 | 0.13 (0.35) | 3.03 (0.32–28.83) | 0.34 |
| Distribution of adverse events | | | | | | | | |
| Delayed cerebral ischemia | 12 | 10 | 0.4 (0.6) | 15 | 12 | 0.5 (0.7) | 1.30 (0.61–2.79) | 0.5 |
| Clinical Seizure | 2 | 1 | 0.07 (0.37) | 2 | 2 | 0.07 (0.25) | 1.00 (0.14–7.10) | > 0.99 |
| Skin Rash | 2 | 2 | 0.07 (0.25) | 1 | 1 | 0.03 (0.18) | 0.53 (0.05–5.81) | 0.6 |
| Bradycardia | 11 | 11 | 0.37 (0.49) | 12 | 12 | 0.4 (0.5) | 1.05 (0.46–2.39) | 0.91 |
| Tachycardia | 8 | 8 | 0.27 (0.45) | 5 | 5 | 0.17 (0.38) | 0.60 (0.2–1.85) | 0.37 |

| All Adverse Events | Galantamine (n = 30) | | | Placebo (n = 30) | | | | |
|---|---|---|---|---|---|---|---|---|
| | Total Events | Patients (n) | Mean (SD) | Total Events | Patients (n) | Mean (SD) | RR (95% CI) | p-val |
| Urinary retention | 2 | 2 | 0.07 (0.25) | 5 | 5 | 0.17 (0.38) | 2.33 (0.45–12.16) | 0.32 |
| Urinary tract infection | 9 | 9 | 0.3 (0.47) | 6 | 6 | 0.2 (0.41) | 0.69 (0.25–1.94) | 0.48 |
| Pneumonia (any) | 11 | 9 | 0.37 (0.61) | 9 | 9 | 0.3 (0.47) | 0.84 (0.35–2.02) | 0.7 |
| Pulmonary edema | 1 | 1 | 0.03 (0.18) | 5 | 5 | 0.17 (0.38) | 4.88 (0.56–42.29) | 0.15 |
| Angio complications (any) | 3 | 2 | 0.1 (0.4) | 1 | 1 | 0.03 (0.18) | 0.36 (0.04–3.46) | 0.38 |
| Surgical complications (any) | 1 | 1 | 0.03 (0.18) | 0 | 0 | - | - | |
| Nausea | 0 | 0 | - | 2 | 2 | 0.07 (0.25) | - | |
| Hyponatremia | 12 | 12 | 0.4 (0.5) | 12 | 11 | 0.4 (0.56) | 1.03 (0.46–2.3) | 0.94 |
| Hypernatremia | 7 | 7 | 0.23 (0.43) | 11 | 11 | 0.37 (0.49) | 1.47 (0.57–3.83) | 0.43 |
| Cerebral edema | 3 | 3 | 0.1 (0.31) | 3 | 3 | 0.1 (0.31) | 1.00 (0.20–4.94) | > 0.99 |
| Anemia | 13 | 13 | 0.43 (0.50) | 12 | 12 | 0.4 (0.5) | 0.91 (0.41–2.00) | 0.81 |
| Pulmonary embolism | 2 | 2 | 0.07 (0.25) | 8 | 8 | 0.27 (0.45) | 3.49 (0.73–16.73) | 0.12 |
| Atrial fibrillation (A-fib) | 0 | 0 | - | 2 | 2 | 0.07 (0.25) | - | |

| All Adverse Events | Galantamine (n = 30) | | | Placebo (n = 30) | | | | |
|---|---|---|---|---|---|---|---|---|
| | Total Events | Patients (n) | Mean (SD) | Total Events | Patients (n) | Mean (SD) | RR (95% CI) | p-val |
| Other | 36 | 19 | 1.2 (1.32) | 34 | 16 | 1.13 (1.36) | 0.89 (0.56–1.44) | 0.65 |

Table 4
Possibility of Adverse Events related to IP

| Possible or Definitely Related to the I.P. | Galantamine (n = 30) | | | Placebo (n = 30) | | | | |
|---|---|---|---|---|---|---|---|---|
| | Total Events | Patients (n) | Mean (sd) | Total Events | Patients (n) | Mean (sd) | RR (95% CI) | p-val |
| Any Adverse Event | 15 | 12 | 0.5 (0.7) | 19 | 17 | 0.6 (0.6) | 1.23 (0.62–2.43) | 0.55 |
| Any Serious Adverse Event | 2 | 2 | 0.07 (0.25) | 0 | 0 | - | - | |
| Adverse event resulting in discontinuation of IP. | 1 | 1 | 0.03 (0.18) | 1 | 1 | 0.03 (0.18) | 0.86 (0.05–14.06) | 0.92 |
| Death during hospital stay | 0 | 0 | - | 0 | 0 | - | - | |
| Distribution of adverse events | | | | | | | | |
| Delayed Cerebral Ischemia | 0 | | | 0 | | | | |
| Clinical Seizure | 2 | 1 | 0.07 (0.37) | 0 | 0 | - | - | |
| Skin Rash | 1 | 1 | 0.03 (0.18) | 0 | 0 | - | - | |
| Bradycardia | 11 | 11 | 0.37 (0.49) | 12 | 12 | 0.4 (0.5) | 1.05 (0.46–2.39) | 0.91 |
| Tachycardia | 0 | | | 0 | | | | |
| Urinary retention | 0 | 0 | - | 5 | 5 | 0.17 (0.38) | - | |
| UTI | 0 | | | 0 | | | | |
| Pneumonia (any) | 0 | | | 0 | | | | |
| Pulmonary edema | 0 | | | 0 | | | | |
| Angio complications | 0 | | | 0 | | | | |

| Possible or Definitely Related to the I.P. | Galantamine (n = 30) | | | Placebo (n = 30) | | | | |
|---|---|---|---|---|---|---|---|---|
| | Total Events | Patients (n) | Mean (sd) | Total Events | Patients (n) | Mean (sd) | RR (95% CI) | p-val |
| (any) | | | | | | | | |
| Surgical complications (any) | 0 | | | 0 | | | | |
| Nausea | 0 | 0 | - | 2 | 2 | 0.07 (0.25) | - | |
| Hyponatremia | 0 | | | 0 | | | | |
| Hypernatremia | 0 | | | 0 | | | | |
| Cerebral edema | 0 | | | 0 | | | | |
| Anemia | 0 | | | 0 | | | | |
| Pulmonary embolism | 0 | | | 0 | | | | |
| Atrial fibrillation (A-fib) | 0 | | | 0 | | | | |
| Other | 1 | 1 | 0.03 (0.18) | 0 | 0 | - | - | |

Table 5

Functional Outcomes, cognitive Outcomes and quality of life

| | Galantamine | Placebo | p-val |
|---|---|---|---|
| modified Rankin Scale median(IQR) | | | |
| 30 day | 3(2–4) | 3(3-4.7) | 0.48 |
| 60 day | 2(2–4) | 3(2–4) | 0.28 |
| 90 day | 2(1–4) | 2(2–4) | 0.37 |
| Montreal Cognitive Outcomes mean(SD) | | | |
| 30 day (n = 51), mean(SD) | 11(10.4) | 13.2(9.5) | 0.5 |
| 60 day (n = 48), mean(SD) | 12.7(10.9) | 12.7(9.8) | 0.6 |
| 90 day (n = 48), mean(SD) | 14(11.5) | 15.8(10.1) | 0.6 |
| EQ5D5L VAS value mean(SD) | | | |
| 30 day (n = 53), mean(SD) | 60(27) | 70(19) | 0.07 |
| 60 day (n = 49), mean(SD) | 71(21) | 69(20) | 0.7 |
| 90 day (n = 49), mean(SD) | 76(19) | 74(23) | 0.76 |
| EQ5D5L Index | | | |
| mean (SD) | | | |
| 30 day (n = 53) | 0.37(0.38) | 0.48(0.39) | 0.24 |
| 60 day (n = 49) | 0.48(0.44) | 0.61(0.36) | 0.29 |
| 90 day (n = 49) | 0.49(0.45) | 0.58(0.44) | 0.33 |

## Figures

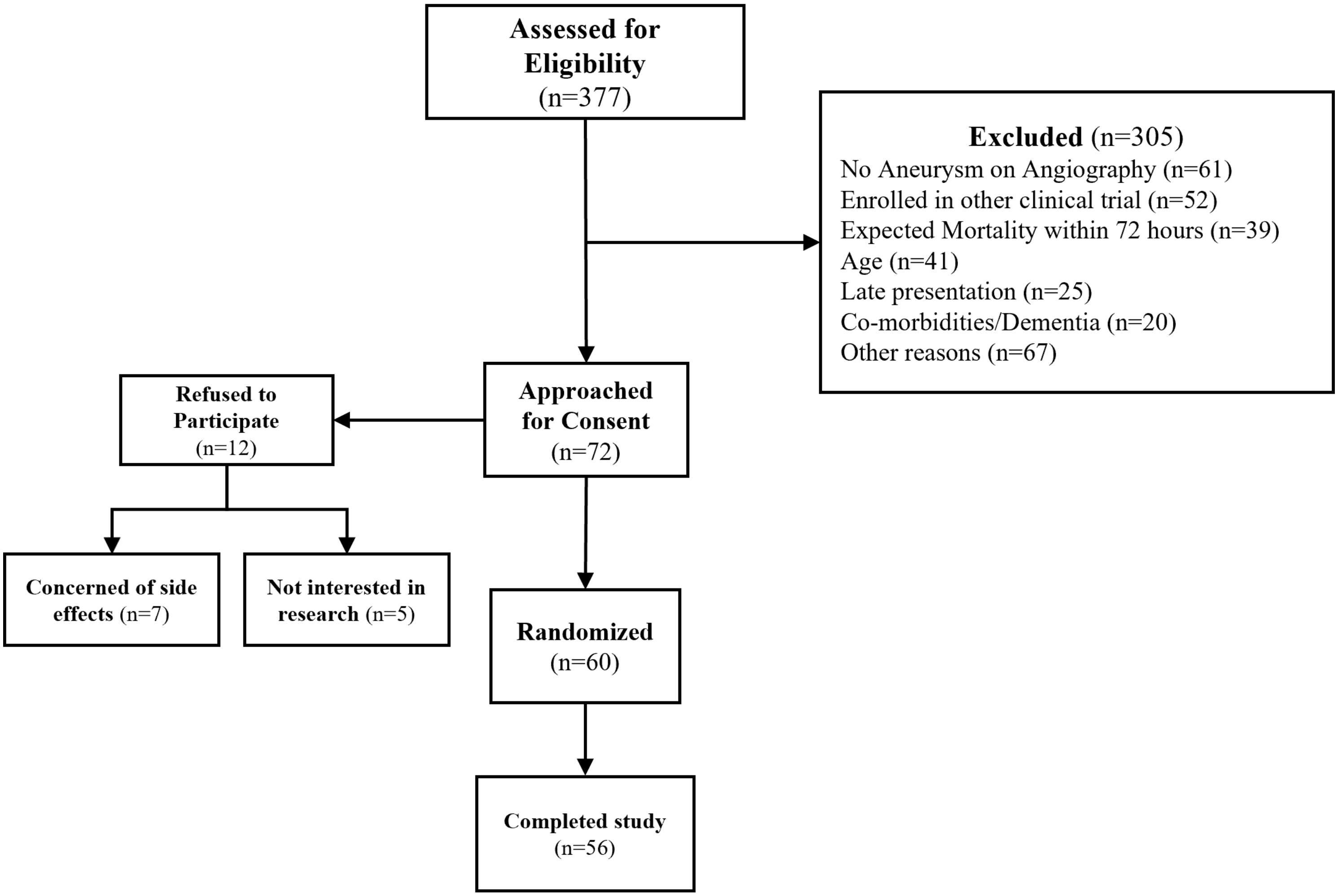

### Figure 1

Flow diagram showing screening, exclusion, consent, randomization, and follow-up design.

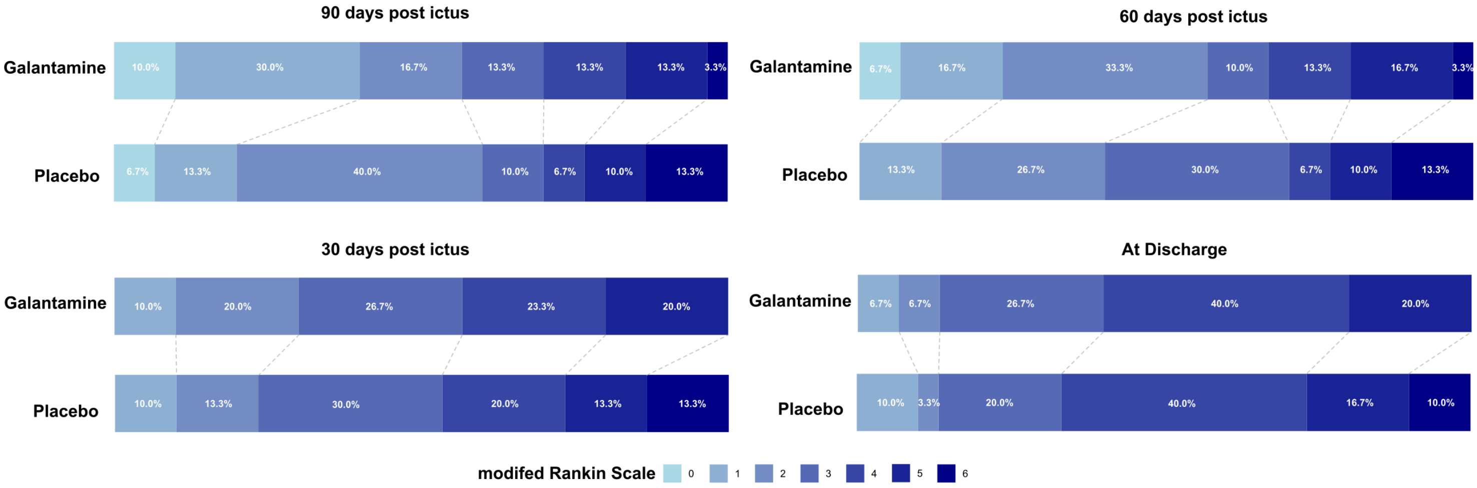

### Figure 2

Distribution of the modified Rankin Scale at different time points

Modified Rankin Scale (mRS) measured at 90 days post ictus did not differ between SAH patients on either galantamine (n=30) or placebo (n=30). Higher proportion of patients in the galantamine group achieved mRS of 0 or 1, compared to the placebo group, but the difference was not statistically significant. Same observations were made at other time points (at discharge, 30, and 60 days post ictus).

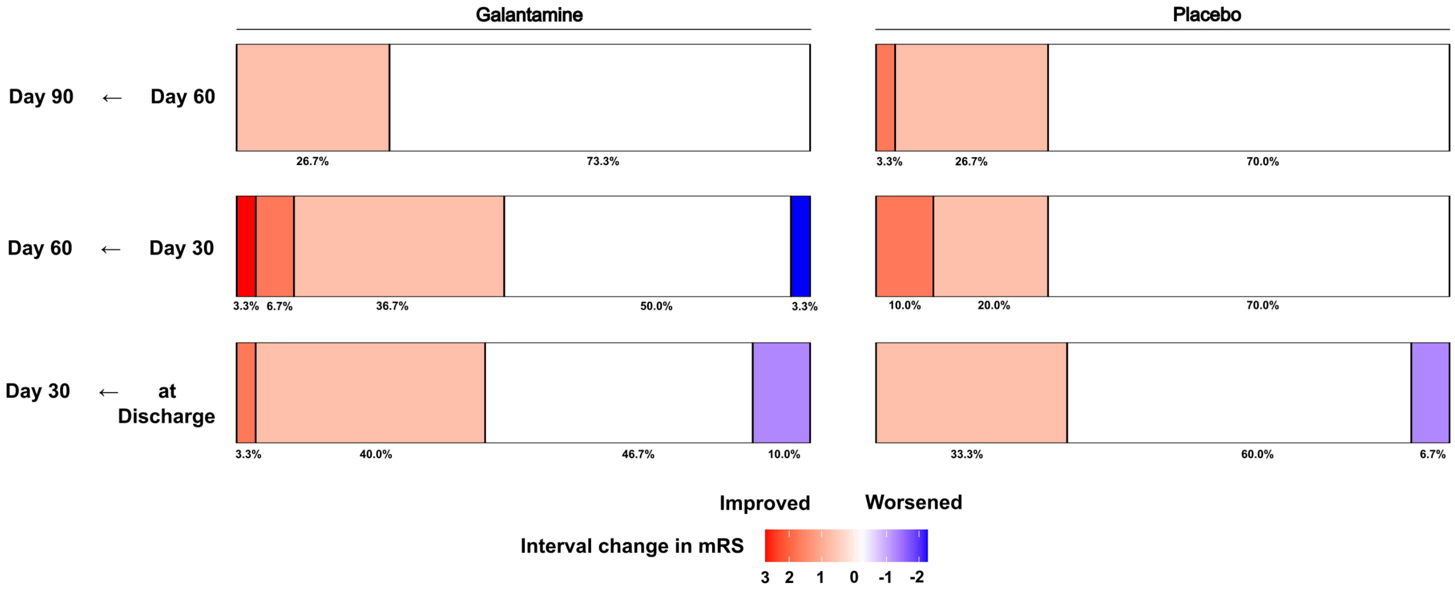

### Figure 3

Comparison of the changes in the modified Rankin Scale between timepoints

Functional improvement, measured with the temporal difference in modified Rankin Scale, was observed in a larger portion of the patients in the galantamine group, compared to the placebo group. The differences were especially large during the earlier stage, within 60 days post ictus. However, all differences were not statistically significant.

## Supplementary Files

This is a list of supplementary files associated with this preprint. Click to download.

- ht14CONSORT2010Checklist20250224v1.doc
- ht14suppfigure120250219.png
- ht14suppfigure220250219.png
- ht14suppfigure320250219.png
- ht14suppfigure420250219.png

- ht14suppfigure520250219.png
- ht14suppfigure620250317.png
- ht14suppfigure720250219.png
- ht14suppfigure820250219.png
- ht14supptables20250317v1.docx
